# Examining the Association between Food Literacy and Food Insecurity

**DOI:** 10.3390/nu11020445

**Published:** 2019-02-20

**Authors:** Andrea Begley, Ellen Paynter, Lucy M. Butcher, Satvinder S. Dhaliwal

**Affiliations:** 1 School of Public Health, Curtin University, Perth 6102, Australia; ellen.paynter@curtin.edu.au (E.P.); s.dhaliwal@curtin.edu.au (S.S.D.); 2 Foodbank Western Australia, Perth Airport 6105, Australia; lucy.butcher@foodbankwa.org.au

**Keywords:** food security, food literacy, cooking

## Abstract

Poor food literacy behaviours may contribute to food insecurity in developed countries. The aim of this research was to describe the apparent prevalence of food insecurity in adults at enrolment in a food literacy program and to examine the relationship between food insecurity and a range of independent variables. Individuals attending the Food Sensations^®^ for Adults program in Western Australia from May 2016 to April 2018 completed a pre-program questionnaire (*n* = 1433) indicating if they had run out of money for food in the past month (food insecurity indicator), frequency of food literacy behaviours, selected dietary behaviours, and demographic characteristics. The level of food insecurity reported by participants (*n* = 1379) was 40.5%. Results from multiple logistic regression demonstrated that behaviours related to planning and management, shopping, preparation, and cooking were all statistically independently associated with food insecurity, in addition to soft/soda drink consumption, education, employment status, and being born in Australia. The results are salient as they indicate an association between food literacy and food insecurity. The implications are that food insecure participants may respond differently to food literacy programs. It may be necessary to screen people enrolling in programs, tailor program content, and include comprehensive measures in evaluation to determine effect on the impact of food literacy programs on different subgroups.

## 1. Introduction

Food insecurity is described as the uncertain or limited physical, social and economic access of individuals and households to sufficient, safe, nutritious, and culturally relevant food [1]. The complexity of this issue is evident in the four pillars (availability, access, utilisation and stability) that underpin the strategies required to achieve food security. Numerous determinants are captured within these pillars including: economic and physical resourcing, food literacy, diet quality and food sufficiency [2]. Whilst there is a focus on poverty and/or the inability to afford food, the lack of or disruption of any of the pillars may ultimately impact food security status [3,4]. Diet-related disease is associated with food insecurity and constitutes a major public health challenge for high income countries [5].

The area of food security attracting less research is the utilisation pillar and an exploration of household or individual food knowledge, skills, and behaviors is warranted [6]. The utilisation pillar incorporates all aspects involved in the safe transformation of food into household meals assuming nutritious foods are available [1]. Encapsulated within utilisation is food literacy, which is described in an Australian food literacy model as the practical food knowledge and skills encompassing the planning, management, selection, preparation, and eating of food [7]. Food literacy contributes to the ability of a person to feed themselves (and others) in a nutrition promoting way [8]. There is a dual relationship between food security and food literacy whereby inadequate food literacy may contribute to food insecurity and being food insecure may limit the ability to use food literacy behaviours to achieve adequate diet quality [9]. Efforts continue to reach agreement on the scope of food literacy and the best way to measure these behaviours. [10]. Research which contributes to our understanding of food security and food literacy relationship will contribute to the evidence base. Food literacy is an aspect that can improve through education and skill development [11] whereas many factors contributing to food insecurity are unmodifiable. A food literate person may be able to develop some resilience to changing personal circumstances, but given the complex reasons for food insecurity it is not the only solution.

Characteristic features of food insecurity in high income countries is the over consumption of high energy foods, reduced intake of fruit and vegetables and limited diet diversity [12,13]. Poor dietary quality has been assumed to be related to a deficit of personal knowledge and skills. For this reason, food literacy programs often aim to improve food security status by building self-efficacy and skills in cooking and budgeting [14]. However, Canadian research has shown that food insecure households do not report lower food preparation skills or cooking abilities when compared with food secure households [15]. The current North American consensus is that the prohibitive income associated with food insecurity does not allow for the implementation of healthy eating principles promoted by nutrition interventions [15,16]. Programs directed at groups experiencing poverty or other high-risk populations such as the Expanded Food and Nutrition Education Program (EFNEP) [17,18] and the Supplemental Nutrition Assistance Program Education (SNAP-Ed) [19,20] show some improvement in the food security status of participants.

Food literacy is thought to improve aspects of food insecurity as improved knowledge and skills may assist to maximise income, but only to a certain point as education cannot change the cost of food or resolve other food insecurity causes that are economic in nature [14,21]. Yet, there is minimal published evidence on the impact of nutrition education and food literacy programs targeting vulnerable populations as many programs are not or poorly evaluated or have small sample sizes [22,23]. Australian program evaluation research shows limited impact [24,25]. There is a need to explore the relationship between food literacy and food security to inform program design and curriculum content. The aim of this paper is to assess the contribution of food literacy behaviours in identification of food insecure participants enrolling in a state-wide government funded adult food literacy program. The objectives were to: (1) describe the apparent prevalence of food insecurity in participants and (2) determine the food literacy, dietary and demographic variables that are independently associated with food insecurity.

## 2. Materials and Methods

### 2.1. Study Design and Recruitment

Participants were recruited for an adult food literacy program from low to middle income areas across Western Australia through existing community groups or advertised public programs. Food Sensations^®^ for Adults (FSA) is a free four-week food literacy program funded by the Department of Health (DoH) WA and run by Foodbank (Western Australia) WA. Originally based on the 1992 FOODcents^®^ key messages [26], the program commenced in 2011. In 2015, FSA underwent extensive revision to align with newly established best practice criteria for food literacy programs [27] based on an Australian food literacy model [7]. The required target group are adults from low to middle income households with low food literacy who want to increase their food literacy skills. Foodbank WA uses a wide range of promotion strategies (including websites, word of mouth, professional referrals, social, and traditional media) to ensure program participants extend beyond typical food bank clientele. FSA is delivered independently from Foodbank WA’s food relief programs. Any West Australian who is able to shop and cook independently is able to enroll in the FSA program. The outcomes of the program required by the funder are to increase the target group’s food literacy self-efficacy, knowledge, and skills, as well as improving food purchasing and preparation in line with the national dietary guidelines. A reduction of food insecurity is not the funder’s expected outcome of the program. As participants are drawn from low income areas it was hypothesized that food insecurity would be above the WA prevalence data.

Individuals (*n* = 2445) attending 212 FSA programs over two years between May 2016 and April 2018 were encouraged where possible to complete a questionnaire before starting the first session. Of these programs 158 were in metropolitan areas (75% of individuals), 54 in regional areas (25% of individuals) as defined by the Australian Bureau of Statistics definition of remoteness [28]. Not all programs were evaluated by facilitators due to decisions on the literacy levels of participants, mental health and disability impacts and not all participants consented to being part of the research process. Participants are not paid for completing the questionnaires. Data analysis was conducted by an independent evaluator and all questionnaire responses were de-identified. This measure was put in place to reduce the incidence of participants responding to questions in a socially acceptable way.

### 2.2. Questionnaire Development

A pre-program questionnaire was developed to address the funder’s required outcomes included a 14-item food literacy behaviour checklist, three food literacy-related practices, four short questions on dietary behaviours and eight socio-demographic variables [29]. The development and validation process for the pre-program food literacy behaviours questionnaire has been published [29]. Questionnaire item selection considered respondent burden, cognitive load and reading levels of participants. The 14 food literacy behaviours were adapted from an extensively tested food behaviour checklist used in EFNEP including one question focused on economic access—“run out of money for food in the past month?”—used as the indicator of food insecurity [30,31,32,33]. Reliability analysis identified three factors: Plan & Manage; Selection and Preparation with high Cronbach’s alpha values 0.76 and above. Eleven of the 13 food literacy behaviours loaded on the three factors using a factor loading cutoff of 0.4 but all loaded using 0.3 as a cutoff point.

Three additional questions on food literacy-related practices were selected from the Department of Health (DoH) WA’s Nutrition Monitoring Surveillance Survey (NMSS), which has been collected every three years since 1998 with a stratified random sample of adults [34]. Two questions on level responsibility for choosing and preparing the household meals and household shopping are similar to those used in the US National Health and Nutrition Examination Survey [35] and one question on self-rated cooking skills drawn from unpublished qualitative research to inform the Go for 2&5^®^ fruit and vegetable social marketing campaign [36]. Four short dietary questions were adapted from the same surveillance survey including two questions on average consumption of serves of fruits and vegetables and two questions on the frequency of consumption of takeaway foods and sugar sweetened drinks. Demographic characteristics collected included sex, age, household composition, highest education level, employment status, postcode, birth in Australia and identify as Aboriginal and/or Torres Strait Islander. For a summary of the questionnaire items, see Table 1.

For the purposes of analysis, the indicator item for food insecurity from the food literacy behaviour checklist was “run out of money for food in the past month?” Food insecurity was determined from participants who indicated running out of food “sometimes”, “most of the time”, or “always” in the month prior to the start of the program (pre-questionnaire). Food secure participants were those who indicated “never” running out of money for food in the past month. The past month time period was chosen to focus on the duration of the program and not the past year as typically is the time frame in national surveys. This question and the one-month time frame were chosen as they have been used in previous program research and evaluation as the key measure of food insecurity [17,21].

### 2.3. Analysis

Statistical analysis was conducted using SPSS (IBM, New York, NY, USA) version 25. Results were considered statistically significant when *p* < 0.05. Pearson Chi-squared tests were used to test for differences in food insecurity status at program entry and exit, and to compare demographic data for those who did and did not answer the food security question. Logistic regression analyses using forced entry method were used to determine whether specific socio-demographic characteristics and food literacy behaviours were predictors of food insecurity. Univariate logistic regression analyses were used to look at each variable independently, each variable was separately regressed on food insecurity status.

Multivariable logistic regression analyses were then carried out to assess 13 food literacy behaviours, three food literacy related practices, four dietary behaviours, and eight socio-demographic variables which together may predict likelihood of food insecurity. Multivariable logistic regression was used to assess the variables associated with binary outcome variable food insecurity. All variables used were coded as categorical. Predominantly the categories used were unchanged from those provided in the questionnaire, however some answers were grouped together for analysis purposes. For household composition Shared house, Supported accommodation, extended family were grouped together. Trade/apprenticeship, certificate/diploma were grouped together in education. Responses to employment status categories were collapsed further; part-time with casual, unemployed with unable to work, and house duties with retired and volunteer. Fruit and vegetable intake was categorized based on the recommended serves from the Australian Dietary Guidelines; whether participants reported two or more serves fruit and five or more serves vegetables daily, or less than recommendations. Income as a primary demographic characteristic was extrapolated from self-reported postcode and converted to the Australian Bureau of Statistic’s Socio-Economic Indexes for Areas (SEIFA) decile ranking of the Index of Relative Socio-economic Disadvantage [37]. Deciles 1 to 7 were considered low-to-middle income and 8 to 10 high-income by methods outlined previously [29]. Food literacy behaviours were recorded to high frequency (most of the time or always) and low frequency (never or sometimes). Food insecurity status was regressed on respondents’ demographic data (characteristics which have previously found to be associated with food insecurity [38]. Then food insecurity status was regressed on food literacy behaviours as well as demographic data to determine relationships between variables. Receiving operator characteristic (ROC) curves were then used for predictive purposes [39]. ROC curves were created for each model to assess sensitivity and specificity to evaluate the predictive ability of food literacy behaviours in addition to other characteristics of participants.

### 2.4. Ethics Approval

Ethics approval was obtained from the Human Research Ethics Committee at Curtin Human Research Ethics Committee (RDHS-52-16). Participants were provided with a verbal explanation of the purpose of the research at the start of their first lesson and a written research information sheet. Written consent was obtained prior to questionnaire administration.

## 3. Results

### 3.1. Response Rate and Level of Food Insecurity

A total of 1433 participants answered the pre-program questionnaire with 1376 (96.1%) responses to the food insecurity indicator question Run out of money for food in the previous month. Of these 819 (59.5%) reported never to this question (food secure group) with the 40.5% reporting sometimes, most of the time or always (food insecure group). Some participants choose not to answer or missed this question despite instructions to complete all questions. Participants who did answer the food security indicator question were not found to be statistically significantly different in any demographic characteristics than those who did not answer the question.

### 3.2. Demographic Characteristics

As reported by participants there was a wide range of demographic characteristics (Table 2). Approximately 80% of program attendees were female, with around 45–50% attendees aged between 26 and 45. Participants reported a wide range of education, 26% and 17% of food insecure participants reported some high school and completed university degree respectively as their highest education compared with 14% and 31% of food secure participants. Of the attendees, 20% of individuals who were food secure reported being unemployed or unable to work, in comparison to 37% food insecure attendees. Participants reported a wide range of household compositions and SEIFA. The majority of participants did not identify as Aboriginal or Torres Strait Islander. Two-thirds of food insecure participants (65%) were born in Australia, compared with 51% of food secure participants.

### 3.3. Food Literacy Behaviours

Responses to the food literacy behaviour questions across the four available answers; “never”, “sometimes”, “most of the time”, and “always” is shown in Table 3. In response to feel confident about managing money to buy healthy food, 41% of food secure participants answered “always”, in comparison to only 9% of food insecure participants. In answering “cook meals at home using healthy ingredients”, 23% of food secure and 15% food insecure participants responded “always”. Just over 10% of food secure attendees answered “never” to “change recipes to make them healthier”, compared with almost 20% of food insecure employees.

### 3.4. Self-Reported Dietary Intake and Cooking Skills

On reporting fast food intake, 32% and 22% of food secure and food insecure participants respectively reported “never” (Table 4). Over half (55%) of food secure participants reported “never” for soft drink intake, compared with just over one-third (36%) of those classified as food insecure. In response to daily fruit intake, 62% of food insecure participants and 55% of food secure participants reported less than two serves. Of the food secure participants, 27% reported they felt they could cook almost anything when rating their cooking skills, compared with 21% of food insecure participants.

### 3.5. Univariate Logistic Analyses

Table 5 presents the demographic results from the analysis and Table 6 the food literacy and dietary behaviours. Both tables present odds-ratio and associated 95% confidence intervals for food insecurity. Analysis is presented as univariate, where variables are singularly, and multivariable, where variables are assessed jointly in its association with food insecurity. Results of two multivariable models are represented, using only demographic variables, and assessing the additional contribution from the inclusion of food literacy behaviours. An odds ratio greater than 1 indicates that the variable is with a greater likelihood to food insecurity. Only statistically significant variables are reported.

Participants were at least 1.7 times more likely to be food insecure if they were under 66 years of age or lived with children or lived alone (Table 5). Participants were also more likely to be food insecure if they were from a low-income area (1.6 times more likely), did not complete a university degree (1.7–3.3 times more likely) or were unemployed/not able to work (2.3 times more likely). Those born in Australia or identifying as Aboriginal or Torres Strait Islanders (respectively 1.8 and 3.1 times more likely) were also more likely to be food insecure. Gender did not significantly correlate with food insecurity.

In relation to planning and managing domain of food literacy, food insecure participants were 1.5 times less likely to “plan meals ahead of time” at the start of the program in addition to “make a shopping list” (1.4 times), “plan meals to include all food groups” (1.4 times), or “feel confident about managing money for healthy food” (1.7 times). When considering the selection domain of food literacy, participants who were food insecure were 1.4 times less likely to “use nutrition information panel for food choices” and “use other parts of food label”. Comparing prices of healthy foods was not significantly different for food secure compared with food insecure participants. Food insecure participants were significantly less likely to “cook meals at home using healthy ingredients” (2.3 times), to “feel confident about cooking a variety of meals” (1.8 times) in addition to “try a new recipe” (1.3 times) or “change recipes to make them healthier” (1.4 times). There was no significant difference between food secure or insecure participants for “thaw meat at room temperature”. There was a statistically significant difference in self-reported cooking skills assessment; more people who were food insecure rated their cooking skills lower. Food insecure participants were 3.3 times more likely to report that they do not or cannot cook rather than being able to cook almost anything.

Participants who were food insecure reported a significantly higher frequency of fast food and soft drink than food secure participants. Food insecure participants were more likely to report having fast food or soft drink on three to four occasions per week rather than never (4.9 and 4.3 times, respectively). Food secure participants were 1.3 times less likely to meet the dietary recommendations for fruit intake (two or more serves per day). There was no statistically significant relationship between individuals meeting the recommended serves of vegetables (five or more serves per day) and food insecurity status.

In Model 2, eight variables were found to be predictors for likelihood of food insecurity (Table 5 and Table 6). These included three demographic characteristics; education, employment and being born in Australia. Participants who were unemployed or unable to work were 2.3 times more likely to be food insecure. Those that did not complete a university degree were at least 1.7 times more likely to be food insecure. Participants who were born in Australia were 1.6 times more likely to be food insecure.

Four food literacy behaviours and one dietary behaviour were associated with food insecurity (Table 5). Participants who did not answer “always” for Feel confident about managing money to buy healthy food were at least three times more likely to be food insecure. Food secure participants were more likely to report they “never” or “sometimes” Compare prices of foods to find the best prices on heathy foods. Participants who reported drinking soft/soda drink more than three times per week were more than 3.6 times likely to be food insecure. Only have some or no responsibility for choosing and preparing the household meals meant a 5- to 6-fold increase in being food insecure. Participants who did not cook or couldn’t cook were 6.3 times more likely to be food insecure.

### 3.6. Multivariable Logistic Regression

**Figure 1:**
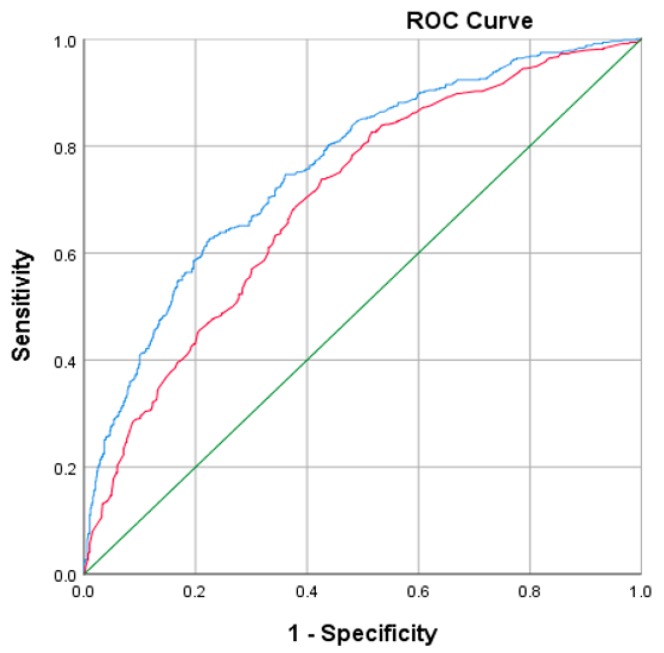
Receiving operator characteristic (ROC) curves to compare the multivariable models ROC curves are represented, using only demographic variables (Model 1 in red), and assessing the additional contribution from the inclusion of food literacy behaviours (Model 2 in blue).

ROC curves were used to compare the two multivariable models. Model 1 (red line) included demographic variables, and Model 2 (blue line) contained additional food literacy behaviour variables (see Figure 1). Area under ROC curve for Model 2 is 0.786 (95% CI: 0.760–0.811) and was significantly greater (*p* < 0.01) than for Model 1 (0.694: 0.664–0.724), indicating the additional contribution of significant food literacy behaviours in identifying subjects with food insecurity.

## 4. Discussion

This is the first Australian study to report on levels of food insecurity in participants attending a food literacy program and a strength of our analysis is the large sample size. FSA is intended for low to middle income participants who may be more at risk of food insecurity but is open to the general population. Our evidence demonstrates an association between food literacy behaviours and food insecurity. Whilst food insecurity is recognised as being underestimated [40], the high level of self-reporting of running out of money for food as an indication of economic access within these participants is concerning. Prevalence of food insecurity in WA shows that one in fifteen adults (6.5%) reported that someone in their household had eaten less than they should because they could not afford enough food in the past 12 months in 2017 [41]. Our results would indicate that food insecurity is much higher in subgroups in the population and the association with employment and education, as indicators of socio-economic status supports this conclusion. The assumption that food insecurity must be higher than national or state prevalence figures in some groups is supported by other researchers who suggest if more sensitive measures were used, food insecurity may be as high as 24.4% in disadvantaged areas [42]. The high level of food insecurity apparent in participants may reflect changing life circumstances at the time of program enrolment such as job loss, divorce or change of household situation producing a negative shift in status [43,44]. Nationally representative data for Australia has documented the independent association of employment (such as sudden job loss) and health (ill health and disability) stressors with food insecurity [45]. The high level of reporting running out of money for food raises concerns for participants in households with children, where there is a strong likelihood of food insecurity impacting on more than just the participants.

The level of apparent food insecurity in participants enrolling in the FSA program is larger than expected given low-income households are not the sole target group. One hypothesis for the high representation of food insecurity is that the cooking and eating component of FSA acts as temporary food relief incentivising vulnerable individuals to attend. Foodbank is the largest hunger relief organization in Australia [46] and in WA delivers a number of nutrition education programs in partnerships with government, industry, and other non-government organisations to at risk groups [47]. Our findings are similar or lower than other nutrition education/food literacy programs targeting vulnerable groups [48]. For example, national data from the American EFNEP shows that of 73,640 participants in 2017, 74% indicated some level of food insecurity as measured by running out of food before the end of the month on program enrolment [49]. After EFNEP program completion this occurred less often for 41% but remained unchanged for 42% and in fact worsened for 17%, which recognises other factors influencing this complex issue and that sufficient food literacy skills are only one aspect needed for to resolve food insecurity. It is possible that participants become more aware of their food insecurity and food literacy skills by the end of the program due to the self-reported nature of the evaluation design.

### 4.1. Food Literacy Domains

All four domains of the Australian food literacy model were associated with food insecurity in this research [7] with the strongest predictors being related to planning, management and selection (responsibility for choosing and planning meals and confidence in managing money to buy food) and preparation and cooking (self-described cooking skills). Poor food literacy is unlikely to be a major reason for food insecurity, our research has shown there were statistically significant differences in most of the food literacy behaviour frequencies and self-described cooking skills for food insecure participants when compared with food secure. It may be that this program does attract people who are food insecure because they perceive they have less skills and/or want to develop their skills.

### 4.2. Planning, Management, and Selection

Responsibility and confidence with planning, management and selection of food are important in food literacy. The ability to prioritise money for healthy food in insecure households will impact on the ability to plan ahead and may result in households changing types of foods to poorer choices. Previous studies have shown that families can be adept at stretching their food dollars and other resources [15,50]. Canadian researchers have documented the ability of food insecure adults to be resourceful and employ a range of strategies to adjust to their situation which may preclude buying less processed healthy foods [15,44,51,52]. In the America there is evidence that less mealtime planning has been associated with food insecurity [53]. In Australia, qualitative research has shown that low-to-middle income Australians face similar challenges in dealing with planning, management and selection of foods with their food security status as just lower income groups [54] indicating programs need to focus on how to prioritise healthy food selection when using planning and management behaviours.

Given the focus on economic access in the measurement of food insecurity, there has been focus on household financial management in research and program delivery. Skills and confidence in general financial management have been correlated with household food security where US households with children with lesser skills and confidence were more likely to be food insecure [54]. The authors concluded this represented a need to improve financial management skills which translated to focusing on food budgeting tips in program design. Huisken, Orr, and Tarasuk (2016) found Canadian food insecure individuals were more likely to have a food budget, but exhibited no other differences in shopping behaviours when compared with food secure individuals [15]. Food selection practices may be important for food insecure families, but the focus should be on more than just food budgeting strategies [55].

### 4.3. Preparation and Cooking

Food insecure participants in our study self-rated lower on cooking skills and this was a predictor of food insecurity. An inverse relationship between healthy food preparation, self-efficacy and severe household food insecurity has been noted in previous research [56]. Regardless, a prevailing theory is that, rather than being a protective factor, food preparation and adaption to a healthy lifestyle are merely inhibited by limited income [56,57]. In contrast to our results, Canadian national results showed no differences in self-rated cooking skills (using different scale to this study) by food insecurity status [15] and the Canadian 2013 Community Health Survey shows the majority of the population self-report having good food skills including meal preparation practices [58]. FSA participants may be self-selecting to attend the program due to perceived skill level as WA state data from the latest 2015 Nutrition Monitoring and Surveillance Series shows higher self-rated cooking skills [35]. Programs need to focus on providing experiential opportunities to develop cooking skills.

Food insecure households have been shown to have similar frequency of cooking but with potentially less complex preparation [57]. Research by Oakley, et al. [59] provides a possible explanation for the comparative simplicity of meals prepared by food insecure people citing that on average food insecure households own fewer cooking appliances. It also is acknowledged that food insecure households may prepare meals that are not considered traditional or reflect societal norms. Crotty, Rutishauser, and Cahill (1992) recognised long ago the unconventional approaches in low income Australian households to feed families using unconventional strategies but also practices such as cutting down on foods perceived as expensive such as fruit and vegetables [60]. More recently, qualitative data has suggested food literacy is an asset as individuals amplify resourcefulness and skills when the budget for food is pressured, therefore providing a buffer when circumstances changed [61].

Establishing associations between cooking, food insecurity and diet quality is limited by the challenge to measuring cooking abilities [62]. The questions used to assess food preparation skills and cooking abilities in this survey have been used elsewhere [29,34], but more research is needed to assess the validity, reliability, and scalar properties of these questions. National surveys have found those spending more time cooking are often from lower income households in the UK [63] and that time use survey data from the US demonstrated less educated women spend more time cooking per day [64] without knowing if this is due to less skills.

### 4.4. Food Literacy Programs as Solutions to Food Insecurity

Food literacy programs are supported as a strategy to reduce food insecurity by policy makers as they focus on the utilisation aspect of food security [65]. This reflects an individual responsibility approach by policy makers to target low income and vulnerable populations with the assumption that these groups are most in need of food literacy development [66]. One of the criticisms of this approach is that programs may fail to reach the intended target group [67] and governments may not commit to addressing the other complex factors for food insecurity such as low welfare income levels, high healthy food prices, and poor food access and availability. Government policy needs to address universal welfare payment adequacy to purchase healthy foods [68] as there is concern that a healthy diet is unachievable for those on welfare [69]. Strategies to address food access and availability are also needed [70].

Food literacy programs are unlikely to ameliorate food insecurity on their own [14]. Our results indicate that it may be necessary to screen participants and tailor program content according food security status in addition to understanding participant’s reasons for enrolment. The American SNAP-Ed program combines supplemental food assistance and nutrition education covering food literacy concepts known as food resource management. Evidence demonstrates that both the supplemental food assistance and improvements in food resource management and are needed to reduce food insecurity in participants as improvements in food resource management alone did not statistically improve food security [19]. Some programs provide targeted produce being featured in recipes on a weekly basis for participants to take home to support behavior change [71]. Where food literacy programs attract a large proportion of food insecure participants their impact is likely to be strengthen when participants receive food relief to improve food security in the short term [72]. Participants’ access to food during and after the program needs to be addressed.

Food literacy programs should be based on theoretical applications to behaviour change by building participant’s self-efficacy resulting in higher confidence to use food literacy behaviours. Higher self-efficacy is thought to improve people’s capabilities across both access and utilisation pillars [73]. It is important that evaluation is comprehensive and sensitive enough to capture the impact of changing food literacy behaviours on those experiencing food insecurity, however evaluation design and measures are considered one of the key limitations of such programs [22,66,74]. Documenting improved food security status will require sensitive measures to be used, such as the validated 18-item United States Department of Agriculture Household Food Security Survey Module [48]. There is an urgent need to develop a validated food security tool for assessment and screening purposes in the Australian context [75]. It is important to establish if food insecurity in participants was episodic or chronic [61]. More research that questions the impact of food literacy programs on different subgroups is needed to contribute to our understanding of the dual relationship between food literacy and food security and ultimately diet quality.

### 4.5. Limitations

There are a few limitations to consider with this analysis and presentation of our results. This is an observation study which can only report on associations between apparent food insecurity and food literacy behaviours and other practices. The recruitment was from participants enrolled in a food literacy program and not randomly sampled from the population. We used one question assessing if participants had run out of money of food reflecting the typical approach used in monitoring and surveillance in Australia to assess food insecurity. This one question relies on self-reported food sufficiency [76] encompassed only within the access pillar and the narrow perspective of the measure has been attributed with underreporting food insecurity in Australia [38,77]. Using additional indicator questions may have resulted in different associations. Not all participants completed evaluation and some participants did not provide an answer to run out of money for food question. Some of the most vulnerable groups and at the greatest risk of food insecurity [78,79] such as people living in remote areas, low literacy populations, and culturally and linguistically diverse groups where not always able to complete the written evaluation and therefore are potentially underrepresented in this analysis. Finally, the generalisabilty of the results to other food literacy programs and populations needs to be considered.

## 5. Conclusions

Food literacy reflects personal behaviours for planning, selecting, preparing, and eating healthy foods and are considered a necessary life skill. Effective programs are needed to build an evidence base approach to program design. Research on the impact of food literacy on food insecurity will assist in program design and confirm if screening those at risk of food insecurity is required. Screening participants for food insecurity prior to entry into the program will enable a tailored approach to education and skill development. Focusing on improving food literacy self-efficacy and skills may help people develop resilience to and manage food insecurity better. Food literacy programs should be delivered in conjunction with food relief to address immediate concerns. At the same time, the four pillars of food security indicate that other strategies and interventions are also required to address the circumstances in which people live and access food. In the long term, advocating for a higher welfare payments or increased government assistance in addition to program provision will provide more tangible results to improving food security.

## Figures and Tables

**Table 1:** Summary of data collected from individuals using the pre- program questionnaire.

Data Collected from Individuals
**Socio-demographic** (8)
Gender
Age
Education
Household Composition
Employment
Postcode
Born in Australia
Identify as Aboriginal or Torres Strait Islander
**Food literacy behaviour** (14)
Occurrence within the last month
Run out of money for food
Plan meals ahead of time
Make a list before you go shopping
Plan meals to include all food groups
Think about healthy choices when deciding what to eat
Feel confident about managing money to buy healthy food
Use the nutrition information panel to make food choices
Use other parts of the food label to make food choices
Compare prices of foods to find the best prices on heathy foods
Cook meals at home using healthy ingredients
Feel confident about cooking a variety of healthy meals
Try a new recipe
Change recipes to make them healthier
Thaw meat at room temperature
**Dietary behaviours** (4)
Weekly consumption of fast food meals, such as burgers, pizza, chicken or chips from fast food outlets
Weekly consumption of regular soft drink (not diet), energy drinks, sports drinks, flavoured mineral water, or vitamin water
Daily intake of fruit
Daily intake of vegetables
**Food literacy -related practice** (3)
Responsibility for choosing and preparing the household meals
Responsibility for doing the household food shopping
Self-described cooking skills

**Table 2:** Demographics of participants attending the Food Sensations^®^ for Adults program between May 2016 and April 2018.

Demographic (*n*)	Response	Food Secure (%)	Food Insecure (%)
**Gender** (1361)	Male	150 (18.1%)	112 (20.2%)
	Female	656 (81.4%)	443 (79.8%)
**Age** (1361)	18–25	94 (11.7%)	88 (15.9%)
	26–35	192 (23.8%)	140 (25.2%)
	36–45	181 (22.5%)	142 (25.6%)
	46–55	93 (11.5%)	92 (16.6%)
	56–65	114 (14.1%)	56 (10.1%)
	66 and over	132 (16.4%)	37 (6.7%)
**Education** (1348)	Some high school	114 (14.3%)	141 (25.7%)
	Completed high school	172 (21.5%)	145 (25.4%)
	Completed TAFE/Certificate/Diploma/Trade	269 (33.7%)	172 (31.3%)
	Completed university degree (undergraduate or higher)	244 (30.5%)	91 (16.6%)
**Household Composition** (1361)	Single person	114 (14.3%)	141 (35.7%)
	Couple with no children	172 (21.5%)	145 (26.4%)
	Single parent with child/children	37 (4.6%)	27 (4.9%)
	Couple with child/children	232 (29.0%)	144 (26.2%)
	Other (e.g., shared or supported accommodation)	244 (30.5%)	92 (16.8%)
**Employment** (1343)	Full time/self employed	100 (12.5%)	55 (10.1%)
	Part time/casual	179 (22.5%)	118 (21.6%)
	Unemployed/Unable to work/disability pension/rehabilitation/prison	157 (19.7%)	200 (36.6%)
	Other: Student/maternity leave/retired/house duties/volunteer	361 (45.3%)	173 (31.7%)
**SEIFA** ^1^ (1309)	Low	306 (39.4%)	251 (47.2%)
	Middle	244 (31.4%)	161 (30.3%)
	High	227 (29.2%)	120 (22.6%)
**Born in Australia** ^2^ (1246)	Yes	381 (50.9%)	325 (65.3%)
	No	367 (49.1%)	173 (34.7%)
**Identify Aboriginal and/or Torres Strait Islander** ^2^ (1234)	Yes	32 (4.3%)	60 (12.2%)
	No	709 (95.7%)	433(7.8%)

^1^ SEIFA, Socio-Economic Indexes for Areas, derived from postcode [38]; ^2^ Added in later version of questionnaire.

**Table 3:** Food literacy behaviours of participants attending the Food Sensations^®^ for Adults programs between May 2016 and April 2018.

Food Literacy Behaviour (*n*)	Response	Food Secure (%)	Food Insecure (%)
**Plan meals ahead of time** (1371)	Never	60 (7.3%)	51 (9.2%)
	Sometimes	364 (44.6%)	287 (51.8%)
	Most of the time	278 (34%)	146 (26.4%)
	Always	115 (14.1%)	70 (12.6%)
**Make a list before you go shopping** (1367)	Never	89 (11.0%)	95 (17.1%)
	Sometimes	234 (28.9%)	175 (31.5%)
	Most of the time	240 (29.6%)	153 (27.5%)
	Always	248 (30.6%)	133 (23.9%)
**Plan meals to include all food groups** (1350)	Never	134 (16.7%)	145 (26.6%)
	Sometimes	332 (41.3%)	218 (39.9%)
	Most of the time	262 (32.6%)	137 (25.1%)
	Always	76 (9.5%)	46 (8.4%)
**Think about healthy choices when deciding what to eat** (1361)	Never	30 (3.7%)	38 (6.9%)
	Sometimes	237 (29.3%)	213 (38.7%)
	Most of the time	411 (50.7%)	223 (40.5%)
	Always	132 (16.3%)	77 (14.0%)
**Feel confident about managing money to buy healthy food** (1369)	Never	42 (5.2%)	67 (12.1%)
	Sometimes	178 (21.8%)	253 (45.7%)
	Most of the time	259 (31.8%)	187 (33.8%)
	Always	336 (41.2%)	47 (8.5%)
**Use NIP to make food choices** (1365)	Never	292 (36.0%)	245 (44.3%)
	Sometimes	332 (40.9%)	209 (37.8%)
	Most of the time	135 (16.6%)	76 (13.7%)
	Always	53 (6.5%)	23 (4.2%)
**Use other parts of food label to make food choices** (1349)	Never	219 (27.4%)	193 (35.2%)
	Sometimes	382 (47.8%)	251 (45.7%)
	Most of the time	149 (18.6%)	74 (13.5%)
	Always	50 (6.3%)	31 (5.6%)
**Compare prices of foods to find the best prices on heathy foods** (1360)	Never	69 (8.5%)	60 (10.9%)
	Sometimes	269 (33.3%)	177 (32.1%)
	Most of the time	300 (37.1%)	195 (35.3%)
	Always	170 (21.0%)	120 (21.7%)
**Cook meals at home using healthy ingredients** (1366)	Never	24 (3.0%)	28 (5.1%)
	Sometimes	160 (19.7%)	193 (34.9%)
	Most of the time	439 (54.0%)	252 (45.6%)
	Always	190 (23.4%)	80 (14.5%)
**Feel confident about cooking a variety of healthy meals** (1364)	Never	46 (5.7%)	52 (9.4%)
	Sometimes	256 (31.5%)	231 (41.8%)
	Most of the time	332 (40.9%)	184 (33.3%)
	Always	178 (21.9%)	85 (15.4%)
**Try a new recipe** (1358)	Never	44 (5.4%)	53 (9.7%)
	Sometimes	442 (54.6%)	308 (56.1%)
	Most of the time	175 (21.6%)	127 (23.1%)
	Always	148 (18.3%)	61 (11.1%)
**Change recipes to make them healthier** (1361)	Never	93 (11.5%)	105 (19.0%)
	Sometimes	439 (54.3%)	300 (54.3%)
	Most of the time	192 (23.8%)	102 (18.4%)
	Always	84 (10.4%)	46 (8.3%)
**Thaw meat at room temperature** (1124)	Never	130 (19.6%)	80 (17.3%)
	Sometimes	218 (32.9%)	164 (35.5%)
	Most of the time	179 (27.0%)	127 (27.5%)
	Always	135 (20.4%)	91 (19.7%)

**Table 4:** Self-reported dietary intake and cooking skills of participants attending the Food Sensations^®^ for Adults programs between May 2016 and April 2018.

Self-Reported Intake and Cooking Skills (*n*)	Food Secure (%)	Food Insecure (%)
**How many times a week on average do you eat fast food meals, such as burgers, pizza, chicken or chips from fast food outlets?** (1222)		
Never	238 (32.4%)	109 (22.3%)
Less than once a week	299 (40.7%)	188 (38.5%)
Once or twice a week	174 (23.7%)	146 (29.9%)
Three to four times a week	16 (2.2%)	36 (7.4%)
Five or more times a week	7 (1.0%)	9 (1.8%)
**How many times a week on average do you drink regular soft drink (not diet), energy drinks, sports drinks, flavoured mineral water or vitamin water?** (1223)		
Never	409 (55.7%)	176 (36.0%)
Less than once a week	170 (23.2%)	113 (23.1%)
Once or twice a week	94 (12.8%)	87 (17.8%)
Three to four times a week	34 (4.5%)	66 (13.5%)
Five or more times a week	27 (3.7%)	47 (9.6%)
**Daily intake of fruit** (1223)		
Less than 2 serves of fruit	407 (55.4%)	302 (61.9%)
2 or more serves of fruit	328 (44.6%)	186 (38.1%)
**Daily intake of vegetables** (1216)		
Less than 5 serves of vegetables	688 (94.0%)	458 (94.6%)
5 or more serves of vegetables	44 (6.0%)	26 (5.4%)
**Responsibility for choosing and preparing the household meals** (1359)		
All	495 (61.3%)	319 (57.9%)
Share	254 (31.7%)	198 (35.9%)
No	57 (7.1%)	34 (6.2%)
**Responsibility for doing the household food shopping** (1360)		
All	458 (56.5%)	316 (57.5%)
Share	292 (36.0%)	194 (35.3%)
No	60 (7.4%)	40 (7.3%)
**Self-described cooking skills** (1360)		
Can cook almost anything	220 (27.1%)	119 (21.7%)
Can cook a wide variety of meals	360 (44.4%)	233 (42.5%)
Can cool a basic meat and 3 vegetables	181 (22.3%)	136 (24.8%)
Can do basic heating of food, use barbeque, boil egg	37 (4.6%)	37 (6.8%)
Can’t cook/Don’t cook	13 (1.6%)	23 (4.2%)

**Table 5:** Demographic variables and its association with food insecurity.

Demographic	Univariate	Multivariable—Model 1 (Demographics Only)	Multivariable—Model 2 (Demographics and Food Literacy Behaviours)
**Age**			
66 and over	1	1	
56–65	1.75 (1.08–2.85) *p* = 0.0233	1.73 (0.98–3.05) *p* = 0.0589	
46–55	3.53 (2.22–5.62) *p* < 0.0001	3.27 (1.88–5.69) *p* < 0.0001	
36–45	2.80 (1.83–4.28) *p* < 0.0001	3.66 (2.19–6.11) *p* < 0.0001	
26–35	2.60 (1.7–3.98) *p* < 0.0001	3.61 (2.15–6.06) *p* < 0.0001	
18–25	3.34 (2.1–5.32) *p* < 0.0001	2.81 (1.6–4.92) *p* = 0.0003	
**Education**			
Completed university degree (undergraduate or higher)	1	1	1
Completed TAFE/Certificate/Diploma/Trade	1.71 (1.26–2.33) *p* = 0.001	1.78 (1.24–2.54) *p* = 0.0015	1.74 (1.13–2.68) *p* = 0.0112
Completed high school	2.26 (1.63–3.13) *p* < 0.0001	2.49 (1.68–3.67) *p* < 0.0001	2.28 (1.43–3.63) *p* = 0.0005
Some high school	3.32 (2.35–4.68) *p* < 0.0001	2.84 (1.85–4.37) *p* < 0.0001	2.05 (1.22–3.47) *p* = 0.007
**Household Composition**			
Other (e.g., shared or supported accommodation)	1		
Couple with children	1.65 (1.2–2.26) *p* = 0.0020		
Single parent with children	1.94 (1.12–3.36) *p* = 0.0188		
Couple with no children	2.24 (1.61–3.1) *p* < 0.0001		
Single person	3.28 (2.33–4.63) *p* < 0.0001		
**Employment**			
Full time/self employed	1	1	1
Part time/casual	1.20 (0.8–1.79) *p* = 0.3781	1.19 (0.76–1.86) *p* = 0.4584	1.07 (0.65–1.77) *p* = 0.7849
Unemployed/Unable to work/disability pension/rehabilitation/prison	2.32 (1.57–3.42) *p* < 0.0001	2.19 (1.4–3.43) *p* = 0.0006	2.28 (1.39–3.77) *p* = 0.0012
Other: Student/maternity leave/retired/house duties/volunteer	0.87 (0.6–1.27) *p* = 0.4723	1.05 (0.68–1.61) *p* = 0.8236	0.76 (0.48–1.20) *p* = 0.2359
**SEIFA** ^1^			
High	1		
Middle	1.25 (0.93–1.68) *p* = 0.1442		
Low	1.55 (1.18–2.05) *p* = 0.0018		
**Born in Australia** ^2^			
No	1	1	1
Yes	1.81 (1.43–2.29) *p* < 0.0001	1.36 (1.04–1.79) *p* = 0.0260	1.61 (1.18–2.19) *p* = 0.0025
**Identify as ATSI** ^2,3^			
No	1	1	
Yes	3.07 (1.97–4.79) *p* < 0.0001	1.3–3.49) *p* = 0.0027	

^1^ SEIFA, Socio-Economic Indexes for Areas, derived from postcode [38]; ^2^ Added in later version of questionnaire; ^3^ Aboriginal and/or Torres Strait Islander.

**Table 6:** Food literacy and dietary behaviour variables and its association with food insecurity.

Food Literacy and Dietary Behaviours	Univariate	Multivariable—Model 2 (Demographics and Food Literacy Behaviours)
**Plan meals ahead of time**		
High frequency	1	
Low frequency	1.45 (1.16–1.86) *p* = 0.0088	
**Make a list before you go shopping**		
High frequency	1	
Low frequency	1.43 (1.15–1.77) *p* = 0.0.0014	
**Plan meals to include all food groups**		
High frequency	1	
Low frequency	1.44 (1.15–1.80) *p* = 0.0016	
**Think about healthy choices when deciding what to eat**		
High frequency	1	
Low frequency	1.70 (1.36–2.12) *p* < 0.0001	
**Feel confident about managing money to buy healthy food**		
High frequency	1	1
Low frequency	3.70 (2.94–4.65) *p* < 0.0001	3.62 (2.66–4.92) *p* < 0.0001
**Compare prices of foods to find the best prices on heathy foods**		
High frequency	1	1
Low frequency	1.05 (0.84–1.30) *p* = 0.6859	0.68 (0.50–0.92) *p* = 0.01
**Use nutrition information panel to make food choices**		
High frequency	1	
Low frequency	1.38 (1.05–1.81) *p* = 0.0.0197	
**Use other parts of food label to make food choices**		
High frequency	1	
Low frequency	1.40 (1.05–1.81) *p* = 0.0132	
**Cook meals at home using healthy ingredients**		
High frequency	1	
Low frequency	2.28 (1.80–2.88) *p* < 0.0001	
**Feel confident about cooking a variety of healthy meals**		
High frequency	1	
Low frequency	1.77 (1.43–2.21) *p* < 0.0001	
Try a new recipe		
High frequency	1	
Low frequency	1.28 (1.02–1.60) *p* = 0.034	
**Change recipes to make them healthier**		
High frequency	1	
Low frequency	1.42 (1.19–1.80) *p* = 0.0.0039	
**How many times a week on average do you eat fast food meals, such as burgers, pizza, chicken or chips from fast food outlets?**		
Never	1	
Less than once a week	1.37 (1.03–1.84) *p* = 0.0327	
Once or twice a week	1.83 (1.34–2.51) *p* < 0.0001	
Three to four times a week	4.91 (2.61–9.23) *p* < 0.0001	
Five or more times a week	2.81 (1.02–7.73) *p* = 0.0458	
**How many times a week on average do you drink regular soft drink (not diet), energy drinks, sports drinks, flavoured mineral water or vitamin water?**		
Never	1	1
Less than once a week	1.54 (1.15–2.08) *p* = 0.004	1.14 (0.80–1.64) *p* = 0.4744
Once or twice a week	2.15 (1.53–3.02) *p* < 0.0001	1.21 (0.79–1.85) *p* = 0.3875
Three to four times a week	4.51 (2.88–7.07) *p* < 0.0001	3.62 (2.66–6.43) *p* < 0.0001
Five or more times a week	4.05 (2.44–6.7) *p* < 0.0001	2.31 (1.27–4.21) *p* = 0.0063
Self-reported fruit intake		
2 or more serves of fruit	1	
Less than 2 serves of fruit	1.31 (1.04–1.65) *p* = 0.0240	
**Responsibility for choosing and preparing the household meals**		
All	1	1
Some	1.035 (0.677–1.58) *p* = 0.874	5.80 (2.74–12.3) *p* < 0.0001
None	0.997 (0.642–1.546) *p* = 0.998	6.46 (3.02–13.83) *p* < 0.0001
**Self-described cooking skills**		
Can cook almost anything	1	1
Can cook a wide variety of meals	1.20 (0.91–1.58) *p* = 0.2047	1.23 (0.86–1.76) *p* = 0.2506
Can cool a basic meat and 3 vegetables	1.39 (1.01–1.9) *p* = 0.0408	0.96 (0.63–1.47) *p* = 0.8477
Can do basic heating of food, use barbeque, boil egg	1.85 (1.11–3.07) *p* = 0.0175	1.46 (0.71–3.01) *p* = 0.3025
Can’t cook/Don’t cook	3.27 (1.6–6.69) *p* = 0.0011	6.38 (1.99–20.43) *p* = 0.0018
